# Enabling Communication Resiliency in the Connected Car Environment

**DOI:** 10.3390/s26041119

**Published:** 2026-02-09

**Authors:** Antonio Guerrero-Ibáñez, Juan Contreras-Castillo, Sherali Zeadally, Een-Kee Hong

**Affiliations:** 1 Faculty of Telematics, University of Colima, 333 University Avenue, Colima 28040, Mexico; antonio_guerrero@ucol.mx; 2 College of Communication and Information, University of Kentucky, 315 Little Library Building, Lexington, KY 40506-0224, USA; szeadally@uky.edu; 3 Academy of Computer Science & Software Engineering, University of Johannesburg, Doornfontein 2028, South Africa; 4 Department of Electronic Engineering, Kyung Hee University, Gyeonggi-do 17104, Republic of Korea; ekhong@khu.ac.kr

**Keywords:** communication, connected car, failures, resiliency, security, vehicular networks, artificial intelligence

## Abstract

Connectivity has become integral to various application domains, with the automotive sector as a prime example. Advances in electronics, computing, and telecommunications have driven the evolution of the connected car ecosystem, transforming it into a data-rich environment that enhances road safety, efficiency, and overall mobility. However, the success of this ecosystem depends on seamless, reliable, and resilient communications. We identify key challenges that may affect communications in the connected car environment and discuss solutions that enhance resiliency and robustness. Finally, we propose a multi-layered network architecture that will enhance communication resilience in the connected car ecosystem.

## 1. Introduction

Urban areas are expanding rapidly. According to the World Bank, approximately 56% of the global population currently resides in urban areas, and this figure is expected to rise to 70% by 2050 [1]. However, rapid and extensive urbanization presents significant challenges in meeting the growing demand for transportation infrastructures to improve traffic conditions, enhance road safety, and reduce pollution levels. The World Health Organization reports that approximately 1.3 million people die, and 20 to 50 million suffer injuries in road traffic accidents worldwide annually [2]. The increasing number of vehicles, traffic congestion, and road accidents accentuate the urgent need for real-time, data-driven decision-making to enhance safety and efficiency for drivers, passengers, and vulnerable road users. Over the past decades, researchers have explored various solutions to address these challenges. These solutions include Intelligent Transportation System (ITS) [3,4,5,6], Vehicular Ad hoc networks (VANET) [7,8,9], and Connected Cars [10,11,12,13,14].

Connected vehicles will play a vital role in the transition of urban mobility systems toward safer, cost-effective and reliable operations, with autonomous services and cooperation among stakeholders. Beyond serving as a means of transport, connected vehicles serve as intelligent nodes and active agents in an urban network. They can exchange data with other vehicles (V2V), roadway infrastructures (V2I), pedestrians, and edge or cloud platforms [15]. Table 1 summarizes the benefits of connected vehicles in the urban transport environment.

### 1.1. Contributions of This Work

In this work, we make the following research contributions:

- We examine the technical challenges that affect communication resiliency in the connected car environment. These challenges include latency, scalability, dynamic topology and data integrity.
- To address the identified challenges, we discuss solutions that will increase the communication resiliency in the connected car environment.
- Based on the state-of-the-art review, we proposed a multi-layer reference architecture that addresses the challenges we identified. It is noteworthy that this proposal represents a conceptual framework. While we validate the architecture through theoretical analysis and asymptotic complexity comparisons, experimental validation via field trials or large-scale testbeds is outside the scope of this paper but will be explored as part of our future work.

### 1.2. Organization of This Paper

We organize the rest of the paper as follows. Section 2 introduces the connected-car ecosystem, outlines its communication modes, and analyzes the associated technical issues. Section 3 discusses the technical challenges affecting communication resiliency in connected car environments and their solutions, including mitigation measures. Section 4 describes the proposed multilayered architecture. Finally, in Section 5, we make some concluding remarks and outline future work.

## 2. The Connected Car Ecosystem

The connected car ecosystem is an environment that integrates various actors that facilitate the operation and interaction of connected cars. The ecosystem actors work collaboratively to enable communication between cars and their operating environment. The ecosystem includes connected cars, road infrastructures, communication networks, service platforms, various technologies, and service providers. This ecosystem focuses on facilitating mechanisms for advanced traffic management, road-crash prevention and traffic congestion and pollution emission reduction. Connected cars create an ecosystem that improves urban planning and provides a basis for integrating new services such as Transportation as a Service (TaaS) and autonomous vehicles, promoting a safer, more reliable, efficient, and sustainable mobility model.

### Communication in the Connected Cars Environment

**Figure 1:**
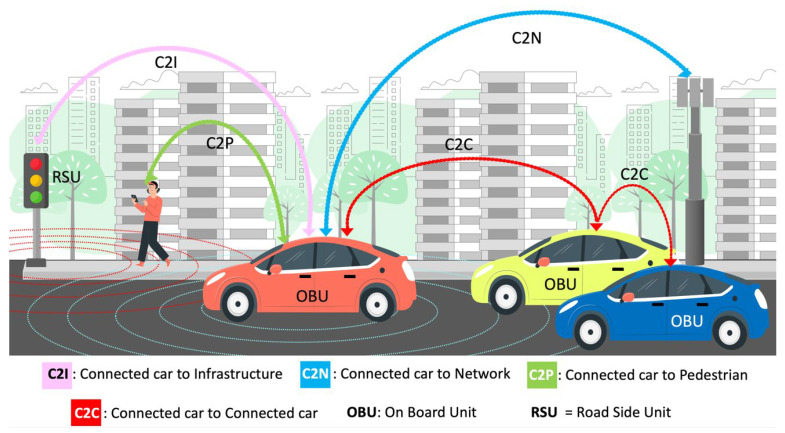
The connected car environment.

Figure 1 presents the different communication modes in the connected car environment. The connected car is equipped with a communication unit, called an On-Board Unit (OBU), which communicates with other network components. Communication with the road infrastructure is enabled through devices (e.g., Road-Side Units (RSUs). In addition, other critical participants in the urban traffic environment include Vulnerable Road Users (VRUs) and communication networks such as cellular systems. Broadly, the interaction among these entities is called Vehicle-to-Everything (V2X) communication. This includes modes such as Car-to-Car (C2C), Car-to-Infrastructure (C2I), and Car-to-Pedestrian (C2P). These communication paradigms enable safe, intelligent, and efficient mobility ecosystems, supporting real-time data exchange among vehicles, pedestrians, and infrastructures. This exchange plays a key role in accident prevention and traffic optimization. However, the deployment of V2X technologies faces numerous technical challenges, which include system interoperability, low-latency communication requirements, continuous real-time coverage, cybersecurity risks, and seamless integration with mobility management platforms.

## 3. Technical Challenges Affecting Communication Resiliency in the Connected Car Environments and Their Solutions

Connected cars require significant attention to resilience challenges because they rely on communication technologies. Sterbenz et al. [16] define resilience as the network’s ability to maintain an acceptable service level under failures and adverse conditions. This concept is especially critical in connected cars, where topology dynamically changes with vehicle density and mobility. In connected-vehicle ecosystems, resilience in safety-critical systems is the capacity to anticipate, adapt to, and recover from faults so that essential functions continue operating. Resilience goes beyond reliability by focusing on behavior under adverse conditions while preserving availability and protecting safety.

Communications in the connected cars environment rely on real-time information exchange between vehicles and road infrastructures to enhance safety, particularly accident prevention, and to optimize traffic operations. However, deployment faces multiple technical challenges (e.g., stringent low-latency and high-reliability requirements, scalability, dynamic topology, and data integrity), as Table 2 shows.

### 3.1. Latency

#### 3.1.1. Challenges

Latency is a key technical issue that can jeopardize the continuity of safety-critical systems; delayed delivery of messages (for example, braking or collision alerts) can lead to crashes with fatal consequences. The ecosystem requires real-time data exchange with tight end-to-end delay bounds across vehicles, infrastructure, and sensors, so that safety functions are not degraded. Mission-critical systems in the connected-vehicle ecosystem require low latency, which conflicts with cryptographic schemes of high computational cost (processing on the order of milliseconds).

Latency creates synchronization gaps between vehicles and infrastructure elements (RSUs, smart signals, control centers). When messages arrive late, connected cars may act on stale data: a braking alert delivered after the fact, a signal phase that flipped seconds earlier, or a pedestrian who is no longer in the crosswalk. High latencies increase risks for drivers and pedestrians and undermine the continuity of safety functions such as collision avoidance, emergency-vehicle priority, and intersection coordination. Operationally, high latency also disrupts the timing required by traffic-flow control algorithms, degrading level of service and potentially amplifying congestion as suboptimal decisions propagate across intersections and corridors.

Latency affects the speed at which the connected car environment adapts to changing network conditions. High delays affect the timely delivery of critical information and limit responsiveness to changing conditions. Latency also directly impacts adaptive control loops, which rely on short sense–decide–act cycles. When the delay is large, decisions are made on stale data, which can destabilize the system rather than improving it. A slow network also hinders instantaneous services (e.g., cooperative maneuvers or emergency priority), making the application unsafe and non-viable. Lowering latency not only improves current performance but also enables new capabilities.

#### 3.1.2. Solutions

Several strategies are used to mitigate latency. Roadside link communications enable direct V2V links with dedicated radio resources, bypassing base stations and reducing latency to 5–10 ms [18,19,20]. Traffic prioritization via QoS and network slicing provisions dedicated “slices” for applications with different profiles (e.g., safety and infotainment) [21,22,23,24,25,26,27]. Multi-access Edge Computing (MEC) moves processing and decision-making to the edge (near base stations), allowing complex machine-learning algorithms [28,29,30,31,32,33], federated learning [34,35], Long Short-Term Memory (LSTM) for vehicle arrival-rate prediction [36], and deep reinforcement learning [37], with reported end-to-end latencies below 1 millisecond. At the physical layer, a technique like Semi-Persistent Scheduling [38] removes per-transmission resource requests, while dynamic rate adaptation to channel quality avoids unnecessary retransmissions.

Mitigation avenues include MAC-layer enhancements, adapting transmission rate and power to vehicular density and applying message prioritization [39,40,41,42]. Hierarchical and clustering architectures can minimize simultaneous transmissions and relieve channel load [43,44]. MEC and hybrid communications, switching between direct C2C and infrastructure-assisted links based on density and channel conditions [45,46] can also be used.

To balance delay and privacy, deployments favor lightweight primitives: ECDSA for message signing and verification with shorter keys and reduced runtimes [47,48,49] and Advanced Encryption Standard in Galois Counter Mode (AES-GCM) for authenticated encryption, providing confidentiality and integrity [50]. In addition, hardware acceleration (for example, crypto engines, AES-NI (Advanced Encryption Standard New Instructions), HSM/TPM (Hardware Security Module/Trusted Platform Module) speeds up cryptographic operations and offloads the main CPUs [51,52].

To improve end-to-end delay, one solution is Multi-access Edge Computing (MEC), wherein computation is offloaded to the edge via RSU-hosted or 5G cell-site servers for events to be processed locally, reducing the number of network hops and round-trip times [53,54]. Additionally, 5G network slicing, combined with Software Defined Networking (SDN) and Network Function Virtualization (NFV), enables isolated logical slices with tailored traffic profiles (such as safety communications with ultra-low latency and high priority), allocating dedicated resources and end-to-end guarantees while keeping non-critical traffic out [55,56,57]. A third solution is proactive prediction where mobility models are trained with federated learning and deployed in the infrastructure to anticipate a vehicle’s trajectory, pre-compute decisions (for example, upcoming signal phase), and transmit the relevant information ahead of time; this minimizes the impact of communication delays and preserves temporal coherence [58]. Together, these measures (edge processing to minimize the number of hops, virtualized networks with strict delay targets, and predictive services) help to keep distributed decisions aligned, limit latency degradation, and maintain the performance of road-safety services.

To ensure low latencies, dynamic traffic prioritization via software-defined networks is employed to identify safety-critical flows and assign them to an ultra-low-latency 5G slice, with preemption and temporary downgrading of non-critical services when congestions occur [59,60]. Another approach is to deploy prediction algorithms using machine learning at the edge. In this case, the models process historical and real-time streams to anticipate changes in vehicle traffic, network demand, and environmental conditions (mobility patterns, channel state, RSU load). Using the forecasts, the system acts proactively: it tunes resource allocation and priorities, schedules retransmissions and paths, adapts modulation/coding, precomputes signal phases, and performs prefetching or caching at nearby nodes. This minimizes latency effects and helps maintain the timely delivery of safety-critical services [59,60,61].

### 3.2. Scalability

#### 3.2.1. Challenges

In dense urban settings, connected cars’ communications may cause packet collisions, channel congestion, and delays, directly degrading reliability and latency. Constant changes in vehicles’ position and speed reshape C2C connectivity and network structure.

Scalability directly affects protection and privacy: as the vehicle fleet expands, the amount of data generated increases, and the identity/certificate lifecycle (issuance, renewal, pseudonym rotation) becomes more complex.

The massive growth of connected vehicles can strain the infrastructure’s communications capacity and computation resources. In congested settings, intensive message exchange leads to an increase in packet collisions and information loss. At the same time, simultaneous data requests from many vehicles can overload MEC servers, causing failures in critical services.

#### 3.2.2. Solutions

To address the adaptability constraints and resource allocation limits imposed by scalability issues, proposed approaches comprise adaptive protocols and tailored variants of Ad hoc On-Demand Distance Vector (AODV) [62,63], Optimized Link State Routing (OLSR) [64,65] and Dynamic Source Routing (DSR) [66,67] which improve responsiveness to mobility. In addition, AI-based predictive models (fuzzy logic, generative AI, machine learning, and deep learning) learn mobility patterns from location and historical traffic data to select more durable routes [68,69,70,71,72]. For sparse traffic, Delay-Tolerant Networks (DTNs) apply store-carry-forward and deep reinforcement learning to buffer data and forward it once a stable connection is available [73,74].

To enable scalability and protect privacy, proposed solutions shifted from centralized management to decentralized issuance of identity or certificate via hierarchical architectures and Decentralized Identifiers (DIDs) [75,76,77]. For certificate revocation, scalable approaches rely on SCMS with batch-segmented CRLs and Bloom filters to speed up checks with low bandwidth usage. Optionally, delta lists and edge-side verification shorten distribution and query times [78,79,80].

Mitigations include adaptive algorithms that tune the transmit rate and power based on congestion levels, with priority given to safety-critical messages [81,82]. Another approach is to form vehicular MEC clusters for load balancing across edge nodes, orchestrated with platforms like Kubernetes to allocate resources and scale instances as demand varies [83,84]. Finally, hierarchical processing (spread across cloud, fog, and edge) offloads upper layers and preserves operational capacity during traffic peaks [85,86].

To address the scalability challenge, microservice architecture and Network Function Virtualization (NFV) enable dynamic horizontal scaling by running Intelligent Onboard System functions as applications. Dynamic horizontal scaling automatically creates additional instances of system functions as vehicular traffic increases, ensuring immediate capacity to process safety data without congestion. The microservices architecture scales only the function experiencing the bottleneck (e.g., obstacle detection), avoiding full-system replication and lowering compute and memory usage. This approach supports Service Function Chaining (SFC) [87] across vehicles and infrastructure elements, with instances that can be instantiated, migrated, or torn down based on network load. In more advanced deployments, node virtualization (vehicles, RSUs, or roadside components) provides a simpler management plane to provision, isolate, and reassign compute, memory, and link capacity without disrupting essential functions [88,89]. A decentralized management approach based on federated learning can also be adopted to leverage on-board data and compute. The environment is partitioned into network regions, and each region trains its own resource-management model to adapt locally (bandwidth allocation, priority queues, function instantiation). Regions coordinate via hierarchical or peer-to-peer aggregation, which lowers core traffic and preserves privacy. These regions are created using asynchronous federated learning with Dynamic Vehicle Selection and Adaptive Aggregation based on Asynchronous Federated Learning (DVSAA-AFL) [90].

Despite its privacy benefits, Federated Learning faces practical deployment challenges in connected car environments due to the ‘Straggler Problem’ and non-Independent and Identically Distributed (IDD) data. In a heterogeneous fleet, vehicles have varying computational capabilities; slower nodes (stragglers) can cause bottlenecks in the global model aggregation process. Furthermore, the rapid topology changes inherent to VANETs mean that a vehicle participating in a training round may disconnect before uploading its gradient update, leading to wasted diverse computational resources and slower model convergence. Consequently, resilient federated learning implementations must incorporate asynchronous aggregation protocols and robust client selection mechanisms that account for link stability, and not just data availability. The scheme updates the AI model weights based on quality, recency, connectivity, and node availability, speeding convergence under mobility and churn while maintaining model performance of intermittent links typical of vehicular networks [90,91]. Finally, density-aware communication protocols, such as Vehicular Position Broadcast (VP-CAST) [92], Urban Multi-hop Broadcast (UMB) [92], and Scalable Broadcast Algorithm (SBA), can be applied so that network behavior adapts to the number of nodes in the area. These adaptive congestion control mechanism schemes tune the beaconing rate, contention windows, transmit power, and modulation/coding to minimize collisions and losses while ensuring timely delivery of safety-critical messages (e.g., beacons and warnings). Urgent alerts are supported through service-class prioritization and differentiated queues, ensuring safety events (e.g., hard braking, pre-collision) are sent with minimal delay even during load spikes. Together, density adaptation and traffic-type priority maintain reliable and scalable communications in vehicular settings [93,94,95,96].

### 3.3. Dynamic Topology

#### 3.3.1. Challenges

The dynamic nature of inter-vehicle links introduces specific challenges: difficulty establishing trust when assessing source reliability, location tracking through trajectory “fingerprints” that erode privacy, and the creation of multiple fake identities. The connected car ecosystem is highly dynamic due to variations in vehicle speed and motion, which makes it hard to maintain stable, trustworthy links. This behavior leads to connectivity loss and dead zones where vehicles lack communications support. The rapid changes in the connected car environment increases chance of missing safety-critical messages from an RSU or nearby vehicles. When vehicles join or leave the connected car environment, the load authentication and signaling tasks also increase.

Adaptation challenges vehicle mobility because the network must constantly adapt and reconfigure to preserve basic connectivity. The topology map (neighbors, links, routes) can become stale within milliseconds, and the high speed of vehicles often splits them into disconnected subgroups. Many adaptations rely on persistent state or active sessions; once a subgroup drops its link, the system cannot guarantee the completion of tasks with long durations.

#### 3.3.2. Solutions

Prior works [97,98] attempted to balance dynamic changes due to mobility and privacy by combining pseudonym schemes (e.g., Modified Merkle Patricia Trie (MMPT), and Merkle Hash Tree (MHT)) with certificateless signatures, making tracking harder while preserving controlled anonymity. To protect basic safety services, software-defined networking has been used to enable centralized, adaptive control, building a global view of the vehicular topology in real time. In this case, the SDN controller can recompute paths, activate backup routes, prioritize broadcasts and timed retransmissions, and coordinate resource assignment across RSUs and access links, aiming to keep critical information delivery on time [99,100]. To achieve this, designs move away from purely reactive routing, discovering paths after each change, toward proactive and position-based approaches that keep stable routes ahead of time. In vehicular settings, Optimized Link State Routing (OLRS)/Destination-Sequenced Distance-Vector (DSDV) (proactive) [101,102] and Greedy Perimeter Stateless Routing (GPSR)/Greedy Perimeter Coordinator Routing (GPCR)/Greedy Traffic Aware Routing (GyTAR) (position/trajectory-aware) [103,104,105,106] are common choices, leveraging GPS coordinates and motion prediction to favor next hops with higher stability. Protocols such as DSR and AODV are reactive; they can be used with extensions (link-quality metrics like Expected Transmission Count (ETX), fast aging timers, multi-path routing, or Delay Tolerant Network (DTN) store–carry–forward strategies) to reduce disconnections and sustain the delivery of safety-critical messages when the network topology changes rapidly [66,96,107]. Other schemes [108,109,110] group vehicles into relatively stable clusters and elect a cluster head based on stability metrics such as speed similarity, distance, and heading alignment (direction of travel) relative to neighbors as well as indicators such as estimated link duration and angular variation. The cluster head coordinates intra-cluster communications, serves as a gateway to other clusters, and minimizes control overhead by preventing all nodes from taking part in network-wide routing. To avoid flapping (rapid oscillation between cluster states or frequent switching of cluster heads), designs use re-election thresholds and hysteresis, with gateway nodes handling inter-cluster traffic as the connected car environment changes.

### 3.4. Data Integrity

#### 3.4.1. Challenges

Data inconsistency affects service continuity: a system built to save lives can become a source of risk when it provides misaligned, stale, or contradictory information. As vehicles concurrently generate and share information, mechanisms are needed to boost system trustworthiness by detecting duplicates, contradictions, delays, or falsified data.

Data consistency directly affects privacy: reconciling discrepancies across various data sources may force the underlying data verification mechanism to disclose more information than necessary. Persistent inconsistency creates vulnerabilities that threaten data protection. The aim is for vehicles to validate an event without exposing their permanent identity. The underlying infrastructure may also yield inconsistent data due to failures of data sources (sensors, vehicles) undermining system trust and safety. Key effects include fusion conflicts when combining data from heterogeneous inputs: subsystem faults, contradictory readings, or vulnerabilities that distort situational awareness and trigger wrong decisions.

When adaptation decisions rely on incorrect or inconsistent inputs, the resulting actions may be useless or harmful: the system adapts to non-existent situations, misinterprets the environment, and makes wrong decisions, eroding trust in its self-management capability.

#### 3.4.2. Solutions

One proposed solution includes trust management systems assigning confidence scores to transmitters based on reporting history [111,112]. Blockchain technology can be used to build immutable, decentralized ledgers that enhance consistency and tamper resistance [113,114,115]. Machine-learning anomaly detection (e.g., physically impossible speeds, inconsistent positions) [116,117,118] and multi-sensor data fusion (LiDAR, cameras, radar, GPS) to cross-validate heterogeneous sources [119,120] are other possible solutions.

Zero-Knowledge Proofs (ZKPs) allow a vehicle to prove message validity and signature correctness without revealing the sender’s identity or auxiliary metadata [120,121,122,123]. This approach helps address data inconsistencies while preserving privacy.

Proposed resilient measures include trust-based data fusion, blockchain, and federated learning to strengthen integrity and privacy. In trust-based fusion, platforms evaluate a source’s historical reputation, proximity to the event, sensor specifications, and consistency with peer observations to determine the trustworthiness of the data originating from various sources. The collected data is then processed with machine-learning methods to tune weights, detect outliers, and reconcile discrepancies, reducing fusion conflicts [119,124,125]. To prevent the tampering of safety-critical messages, blockchain-based trust management systems employ distributed ledger technology: each transmission is recorded immutably, which hinders manipulation and provides an auditable trail for post-event analysis [126,127]. However, applying blockchain in high-mobility V2X scenarios introduces significant trade-offs regarding latency and scalability. Traditional consensus mechanisms (e.g., Proof-of-trusted-work [128]) incur confirmation delays that far exceed the 10–100 ms safety margins required for critical messages (such as pre-crash warnings) [129,130]. Even with lightweight, permissioned ledgers, the storage overhead and cryptographic verification time on resource-constrained On-Board Units (OBUs) can degrade system performance during high-density traffic events. Therefore, for a resilient architecture, blockchain is best suited for the asynchronous control plane (e.g., identity management, liability attribution) rather than the synchronous data plane responsible for immediate safety maneuvers.

Additionally, Federated learning improves perception models without sharing raw data: each vehicle and RSU trains on its own samples locally and then sends only model updates (parameters and weights) to an infrastructure server, where a global model is aggregated. This approach limits exposure of sensitive information and helps mitigate inconsistencies across sources. To address this challenge, reputation and trust models assign a score to each node (vehicle or RSU). The score is then updated from its accuracy history, spatiotemporal consistency with neighboring reports, proximity to the event, and false-alarm rate, with weights. Based on that score, the system filters dubious sources, adjusts the weights in data fusion, applies thresholds to quarantine nodes, and triggers grey/blacklists. To curb Sybil attacks, cryptographic verification and multi-source consensus are required before accepting safety-critical events [131,132].

It is worth noting that we recognize that to comprehensively evaluate the impact of the proposed solutions for the identified challenges, we need a dedicated testbed that can evaluate both their effectiveness on communications in a connected car environment. This evaluation is beyond the scope of this work, but we will develop such a testbed as part of our future work.

Resilience in connected-vehicle environments rests on a set of axes spanning technical dimensions. These axes determine the capabilities needed to maintain continuity of critical functions. Operational continuity means keeping services such as collision warnings, traffic control, and pedestrian interaction available always, with controlled degradation during incidents. This requires network traffic prioritization (Quality of Service (QoS)), low-latency, high-reliability protocols, link and path redundancy, and rapid failover mechanisms.

In the connected-vehicle ecosystem, systems generate a large amount of sensitive data such as location, mobility patterns, driver behavior, and even personal interactions. This concentration of data makes the ecosystem an appealing target for theft or misuse. To keep privacy and protect data even under compromised conditions, the ecosystem should incorporate advanced, consistent measures such as encryption for data in transit and at rest, access control, and data minimization or anonymization.

The connected-vehicle ecosystem comprises a heterogeneous infrastructure that may expose vulnerabilities if components do not communicate securely or if interoperability is limited. A resilient environment should support open standards, modular architectures, and the co-existence of multiple communication technologies (DSRC, C-V2X, vehicular Wi-Fi) to ensure service continuity.

Adaptability refers to the ability to adjust behavior and operations when conditions change, whether due to rising node density or component failures. The ecosystem must adapt to variations in network and communication conditions, treating them as opportunities to improve performance.

**Figure 2:**
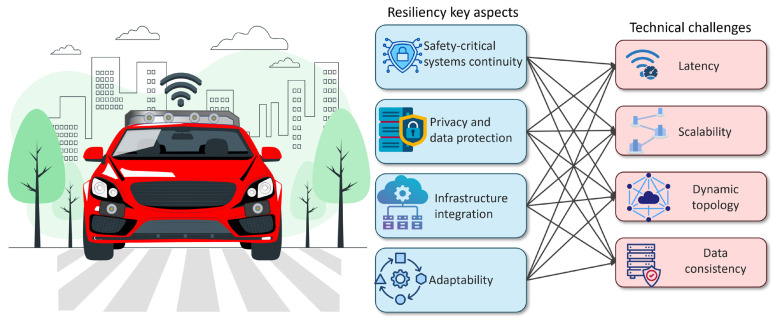
Shows the key resiliency aspects and the associated technical challenges.

The connected-vehicle ecosystem must ensure safety and trust for users. Drivers, pedestrians, and system operators should perceive the vehicular environment as safe, reliable, and respectful of privacy. Failures can hinder adoption regardless of technical merits of the connected car environment. Table 3 summarizes key aspects that highlight the importance of resilience in a connected car domain. Figure 2 shows the key aspects and the associated technical challenges graphically.

### 3.5. Survivability

In this work, we distinguish between resiliency and survivability in the connected car environment. While resiliency ensures the maintenance of acceptable service levels under adverse conditions, survivability specifically focuses on the system’s ability to maintain a subset of essential services (e.g., basic braking) during a failure event, even if non-critical functions are unavailable.

For decades, a foundational design principle in communication networks has been network survivability [133]. This principle emphasizes sustaining connectivity and services during common predictable failures (link cuts, node outages).

In VANETs, the survivability paradigm is, at best, insufficient and, at worst, irrelevant. In vehicular settings, failure is not an exception; it is the operating condition. Network topology changes occur so quickly in the connected car environment at times that any precomputed backup path becomes stale within milliseconds; thus, we need to shift from path-centric survivability to information-centric survivability.

In traditional models such as connection-oriented survivability models in packet-switched networks [134] and traditional network survivability and resilience frameworks [135], survivability is measured by connection restoration. In VANETs, V2V link loss due to mobility, blockage, or interference is common and does not, by itself, constitute a system failure. A vehicle leaving a road because it turns is not a failed node. Accordingly, we define failure as the system’s inability to deliver critical data (for example a collision alert) to the relevant nodes within the appropriate timeframe.

Traditional network survivability relies on three basic principles, protection, restoration, and redundancy, which remain valid in VANETs, though they call for a different realization. Classical protection reserves backup resources. However, in vehicular networks, static reservation is impractical; an analogous approach is proactive data redundancy. Rather than sending a packet along a primary path with a backup path, survivability is achieved through opportunistic dissemination and epidemic routing, allowing multiple transient routes to deliver information to the intended receivers. Network coding provides a strategy that better withstands intermittent links: nodes combine packets, and any vehicle that collects enough coded blocks, regardless of origin, can reconstruct the original content. This method tolerates loss more effectively than simple retransmission by decoupling delivery from a single path and leveraging the spatial–temporal diversity of the vehicular environment.

Classical restoration recomputes a path after a failure. In VANETs, this is too slow because reactive protocols converge more slowly than mobility evolves. Survivability is better achieved through connectivity prediction, anticipating link degradation and preparing handoff before it happens. A key point is that vehicles are not random movers; they follow road layouts, exhibit traffic patterns, and obey physical constraints. This structure can be leveraged with GPS, digital maps, and density measurements to build predictive models of link availability. With such forecasts, the resilient protocol favors the highest-stability path over the shortest path for the required exchange window. Key cues include relative speed and heading alignment (the vehicle’s direction of travel relative to the destination), proximity to intersections and likely turns, received-signal trends (analyzing RSSI patterns over time rather than just instantaneous strength), link expiration time, and near-term capacity. Using these estimates, the system performs pre-routing, proactive handovers, timed multipath, and opportunistic forwarding to complete data transfers even as neighborhoods change.

Pure V2V networks are inherently stochastic, and adding infrastructure such as RSUs and MEC reintroduces stable nodes. These elements act as islands of stability or survivability anchors, providing persistence and coordination capability. In low-density areas, for example rural highways, V2V connectivity is intermittent. In such settings, RSUs serve as anchors or restorers by storing and retransmitting safety-critical messages, bridging connectivity gaps between sporadic vehicle encounters. The MEC edge enables buffering, aggregation, and scheduled forwarding within validity windows so that safety information remains available until it reaches relevant receivers. This survivability can be orchestrated centrally or in a federated fashion by SDN controllers at the edge. Core tasks include dynamic radio-resource assignment via slicing, prioritization of safety flows over non-critical traffic, differentiated queuing, and proactive handover when link degradation is predicted. Together, these measures ensure the timely delivery of essential messages even under heavy congestion or topology fragmentation.

Traditional survivability metrics such as availability and Mean Time to Recovery, do not fit VANETs. Assessment must be service and information-centric: a network is survivable if it sustains a low Age of Information (AoI) for critical applications despite the rapidly changing network topology.

Harnessing survivability in VANETs calls for a paradigm shift: systems must embrace intermittency. Survivability is not achieved by shielding physical links, but through a cross-layer design that blends data redundancy at the application or transport layers, predictive network-level models to forecast topology, and infrastructure support acting as a resilience buffer and resource orchestrator.

## 4. Proposed Resilient Architecture for the Connected Car Environment

**Figure 3:**
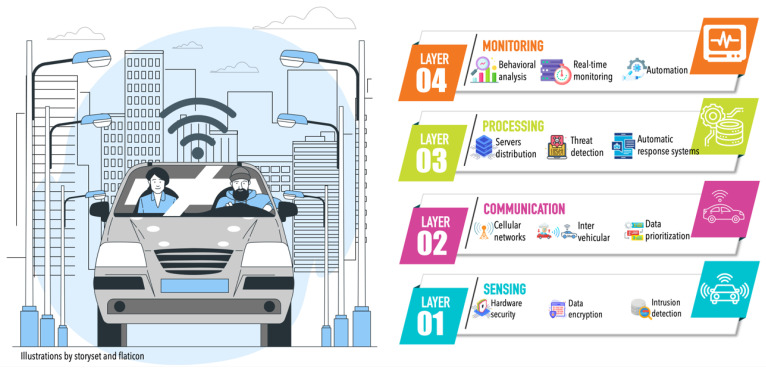
Proposed four-layer resilient architecture for the connected car environment.

For the connected car environment to achieve and maintain resilient communication, we must identify and anticipate potential failures and provide robust solutions that will ensure that the connected car environment can withstand and quickly recover from such failures when they do occur. We propose an architecture based on a four-layer (comprising sensing, communication, processing, and monitoring layers) model (as Figure 3 shows) that provides resilient communication in the connected car environment. Our proposed architecture supports multiple functions, which include security, redundancy, and automated response in connected cars.

### 4.1. Sensing Layer

This layer collects all the data from the environment to monitor communications and detect possible failures within the environment to make real-time decisions and adapt to changing conditions. This layer ensures the reliability, integrity, availability, and security of devices and sensors inside and outside the vehicle. The layer must ensure that all data generated or exchanged within the connected car environment is secure, accurate, and available and does not suffer integrity issues due to tampering.

The sensing layer employs an array of sensors to collect various types of data from the surroundings, addressing each sensor’s unique performance issues under various weather conditions to ensure the connected car environment operates seamlessly. Sensor redundancy mechanisms are deployed in critical or conflict-prone areas within the driving environment to compensate for any individual sensor deficiencies. This redundancy is crucial for validating data integrity and guarantees the acquisition of pertinent information even when a sensor malfunctions. Moreover, this layer design contains adaptive functionalities that dynamically adjust sensor settings, such as configuration or sampling rates, in response to environmental factors such as weather, lighting conditions, and traffic density. Data fusion techniques enhance accuracy and reliability by synthesizing information from multiple sources, which helps develop a comprehensive and accurate overview of the connected car ecosystem. Additionally, the layer incorporates advanced communication protocols to facilitate rapid and dependable data exchange among sensors and the infrastructure critical for supporting low-latency, real-time services and enabling timely decision-making in dynamic settings.

This layer must implement functions that ensure data integrity and confidentiality internally and externally to the car. Data encryption mechanisms can be implemented for communications both inside the car systems and outside the car when exchanging information with other elements of the environment (other cars, infrastructure, or pedestrians). It must implement strong authentication functions for devices to allow only authorized sensors to send data to the control systems. It must also implement mechanisms to detect and mitigate cyberattacks to protect all car control systems and prevent physical tampering.

### 4.2. Communication Layer

The communication layer facilitates efficient and reliable data exchange among vehicles, pedestrians, and infrastructure in the connected car ecosystem. This layer supports communications between cars (C2C), between cars and infrastructures (C2I), and between cars and pedestrians (C2P). This communication is associated with many challenges to ensure secure, resilient, and adaptive communication in a connected car environment. Integrating various communication technologies to provide reliable, safe, and efficient communication is imperative. These encompass wireless local area networks, in-vehicular networks, and state-of-the-art cellular technologies such as 5G or 6G, critical for extending connectivity coverage, providing high bandwidth, and minimizing communication latency. Technological diversity will facilitate seamless service operation with a strong focus on user safety.

Moreover, all components of the connected car environment must have embedded security measures, including the communication systems and vehicles’ onboard computers, the infrastructure, and any associated devices. These security measures should support advanced encryption, intrusion detection systems, and continuous monitoring capabilities to protect against unauthorized access and cyber threats. By doing so, we can preserve the integrity and reliability of the communication channels and ensure the privacy and safety of all users. This layer uses secure communication protocols, such as Elliptic Curve Cryptography (ECC), Transport Layer Security/Secure Sockets Layer (TLS/SSL), or blockchain, to establish encrypted connections for all modes of communication [136,137,138,139], along with digital signature mechanisms to ensure that messages exchanged in the connected car environment cannot be tampered [139,140,141]. Furthermore, strong encryption enables the secure coordination between vehicles. It is essential for transmitting and receiving real-time safety alerts, which are integral to the proactive safety mechanisms of connected cars.

This layer also incorporates advanced infrastructure redundancy management mechanisms for enhancing the reliability, availability, and security of communications among vehicles, pedestrians, and infrastructure. Deploying multiple access points and base stations to achieve a resilient network can provide infrastructure redundancy, particularly in dense urban areas with a strong road infrastructure in place, to guarantee uninterrupted service delivery even in the presence of failures (e.g., access point failure). Facilitating alternative data transmission paths is crucial for maintaining continuous communication capabilities in the connected vehicle environment, ensuring resilience against disruptions caused by electromagnetic interference, network congestion, changes in topology, or other unforeseen issues. Furthermore, dynamic routing protocols are necessary to address vehicular networking challenges such as dynamic topologies, varying congestion levels, low latency, and data transmission overhead. Equally important is implementing priority mechanisms for critical data streams, such as security alerts or emergency messages, to make sure that urgent information is transmitted promptly, securely, and reliably.

Lastly, integrating dynamic bandwidth management mechanisms [142,143,144] is critical for optimizing data flow during transmission. This can involve innovative solutions such as data compression and adaptive image resolution reduction, which are particularly useful in scenarios with high network traffic. Such measures ensure that information exchange is successful and efficient, maintaining service continuity even under congested network conditions [145,146].

The proposed architecture is designed with forward compatibility for 5G-Advanced and 6G ecosystems. Specifically, the Sensing and Communication layers are structured to support Integrated Sensing and Communication (ISAC), where radio signals serve the dual purpose of data transmission and radar-like environmental perception. Furthermore, the Processing Layer is ready to host AI-native air interface algorithms, allowing the network to use machine learning not just for the application logic but also to dynamically optimize physical layer parameters (e.g., modulation and coding schemes) in real-time, to meet the Ultra-Reliable Low-Latency Communication (URLLC) requirements of future autonomous platooning.

### 4.3. Processing Layer

This layer is critical for analysis and decision-making based on sensor and communication channel data. It supports the processing, storage, and seamless availability of services in the connected car environment. This layer’s architecture is highly distributed, scalable, and fault-tolerant to strengthen resiliency in the connected car ecosystem. It supports a redundant and high-speed connectivity framework that evenly distributes processing loads across numerous vehicular and infrastructure nodes, enhancing resilience by ensuring that a single node failure does not compromise the system’s overall functionality.

Moreover, the architecture supports robust, secure storage solutions for all sensitive data, and uses advanced data encryption and access control mechanisms to protect against unauthorized access and ensure data integrity. Architectures that integrate fog (using zonal and cluster-based architectures) and edge computing into the vehicular environment have been developed for data security and privacy and to reduce processing overhead due to the lack of resources needed for information processing. Edge computing is used for data processing directly in the vehicle, reducing latency, a relevant metric for mission-critical applications such as autonomous driving or collision avoidance systems [147,148]. In addition, reducing the need for transmission of data to the cloud data improves privacy. Fog computing is implemented in road infrastructures such as traffic lights, communication towers, and others, which are responsible for storing and processing data from vehicles circulating in the environment, thus facilitating the coordination of vehicles in the road environment. In addition, since the processing is distributed across multiple nodes, the load on the central cloud is reduced, and the scalability of the system is improved [149,150,151]. To complement this distributed architecture, the vehicles and infrastructures should have processing capabilities that handle essential tasks autonomously, even during network disruptions. This capability significantly increases resilience, particularly in areas with fluctuating network connectivity. A set of infrastructure nodes is defined as a monitoring node. The designation depends on the node’s location and the routing protocol. The monitoring nodes apply advanced data fusion algorithms to enhance the accuracy and dependability of data analytics, which are vital to the functionality of mission-critical applications such as collision avoidance, real-time emergency notifications, remote driving assistance, and adaptive cooperative cruise control in the connected car ecosystem.

Additionally, this layer incorporates intelligent mechanisms to dynamically prioritize processing activities based on the data’s urgency and significance. It supports features that adaptively manage processing resources in response to varying network conditions and system demands. This strategy significantly impacts the effectiveness and resilience of ongoing service operations. Workload-adaptive cloud services [145,146,147] can guarantee continued data access even during local processing failures. Adaptive workload services are a category of cloud services designed to dynamically adjust resources and capacity according to the specific needs of workloads in real time. These services enable cloud providers to optimize performance, efficiency, and costs by intelligently allocating resources based on current user demand. In this way, computing, storage, and network resources can be automatically scaled according to varying user demands, optimizing resource usage to minimize costs and maximize operational efficiency.

In the processing layer, virtual machines deployed in roadside infrastructures execute applications in isolated and scalable environments, and containers (such as Docker) promote portability and resource efficiency by encapsulating applications and dependencies [148]. Load balancing algorithms must distribute different service requests among multiple servers (using models such as fog computing, edge computing, and cloud computing) to avoid overloading, service disruptions and reduce service latency.

While standard blockchain consensus mechanisms (e.g., Proof-of-trusted-work) introduce high latency [149], the proposed architecture employs a lightweight, permissioned ledger system strictly for non-real-time tasks, such as identity management and post-event liability auditing. Critical safety messages (e.g., collision warnings) bypass the ledger and are transmitted directly via the Communication Layer using fast cryptographic primitives, Elliptic Curve Digital Signature Algorithm (ECDSA), to ensure millisecond-level latency. The blockchain serves as a secondary, asynchronous layer to maintain an immutable trust score for each vehicle without impeding immediate safety operations.

To address the limitations identified in Section 3, our Processing Layer implements a ‘Split-Path’ strategy. Computationally intensive tasks with strict latency requirements (e.g., collision avoidance) are processed via lightweight edge logic, bypassing heavy consensus protocols. Conversely, non-time-critical tasks (e.g., model training via federated learning or trust score updates via Blockchain) are offloaded to asynchronous background processes. This design ensures that the overhead of these advanced technologies does not compromise the survivability of essential safety functions.

### 4.4. Monitoring Layer

The monitoring layer plays a vital role in safeguarding operational integrity, ensuring service continuity, optimizing performance, and strengthening the security of the architecture in the connected car environment. The primary function of this layer is to manage and monitor the environment’s infrastructure and services. This layer must identify and detect potential problems in real-time and efficiently respond to those events to ensure the continuous operation of the connected car environment.

#### 4.4.1. Real-Time Monitoring

To effectively implement this layer in a resilient, connected car environment, comprehensive real-time monitoring capabilities must be integrated into the vehicles and the surrounding road and network infrastructures to continuously monitor and report on the status of connectivity, network load, infrastructure status, and the performance of all critical services and systems. The insights gained from this real-time information are crucial for promptly identifying issues such as hardware malfunctions, network bottlenecks, or traffic irregularities. This proactive monitoring enables the immediate execution of corrective measures, including the automatic deployment of backup systems, strategic traffic rerouting, or the deployment of specific mitigation strategies. Such rapid responses are needed to prevent any degradation in system performance, thus guaranteeing uninterrupted service delivery and maintaining the connected car environment’s overall efficacy.

#### 4.4.2. Warning Systems

The connected car environment and its service performance can be analyzed in real-time using collected data on network latency, resource allocation, and errors to detect potential problems early on and ensure service continuity. This layer proactively manages traffic changes to preemptively address problems that could lead to critical failures. It incorporates advanced early warning systems to promptly alert about events that might disrupt the operation of the environment. Supporting all these functionalities is vital for ensuring the resilience of services offered in the connected car environment.

#### 4.4.3. Automatic Alert Management

This layer includes automatic alert management modules based on predefined thresholds for critical metrics such as vehicle speed, following distance, weather, and road conditions. It also enables a proactive response system which is invoked before potential issues substantially degrade the performance of the services and the environment.

#### 4.4.4. Auditing

This layer includes comprehensive auditing capabilities for an in-depth log analysis and adherence to stringent security requirements. Auditing is vital in identifying unusual activities, configuration changes, software updates, or potential security breaches. These insights play a pivotal role in increasing the resilience of the connected car environment. Additionally, the auditing process is also instrumental in the ongoing improvement of the connected car environment. Through process optimization, security enhancement, and preemptive measures against cyber threats, we can further improve the resilience of the connected car environment.

### 4.5. Cross-Layer Interaction and Data Flow

From an implementation perspective, the interaction between the four layers is governed by defined Service Access Points (SAPs) to ensure modularity. The Sensing-to-Communication interface encapsulates raw sensor data into standardized Basic Safety Messages (BSM) following the Society of Automotive Engineers (SAE) J2735 protocol [150], ensuring compatibility with DSRC and C-V2X stacks. For internal vertical communication (e.g., from Sensing Layer to Processing Layer within the vehicle), the architecture proposes the use of high-performance Google Remote Procedure Calls (gRPC (https://grpc.io/) [151] or shared memory buffers for real-time data, thereby avoiding the serialization overhead of traditional REpresentational State Transfer (REST) Application Programming Interfaces (APIs). Conversely, the Monitoring Layer interacts with the control plane via the asynchronous Message Queuing Telemetry Transport (MQTT) protocol [152], allowing it to subscribe to system health metrics (‘heartbeats’) without blocking the critical data path.

**Figure 4:**
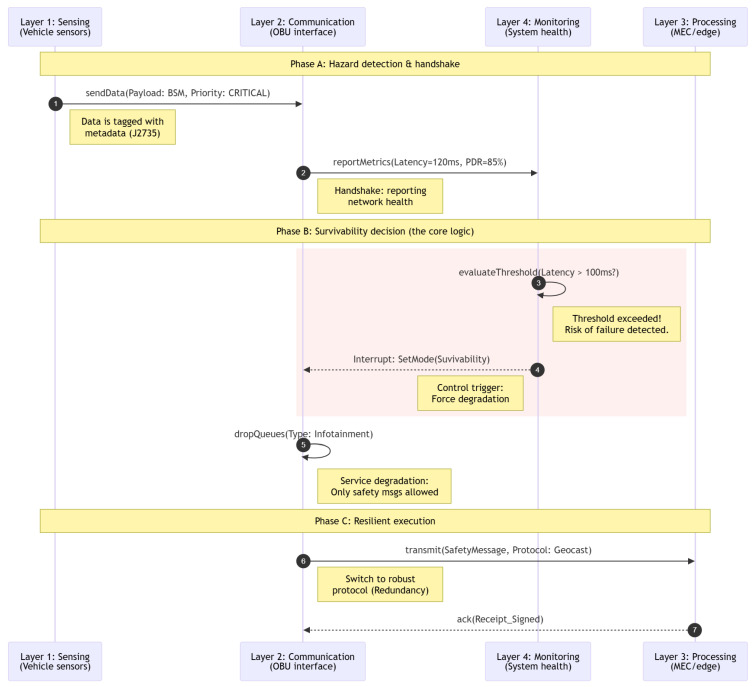
Sequence diagram showing the cross-layer handshake and the activation of the survivability mode.

Figure 4 illustrates the operational data flow during a safety-critical event (e.g., obstacle detection), explicitly defining the inter-layer interfaces and decision-making logic designed to ensure resiliency and low latency. The process is orchestrated across the four proposed layers as follows:

Figure 4 presents the specific cross-layer protocol and decision logic used to activate the ‘Survivability Mode’ during a network hazard. As the sequence diagram shows, the process is orchestrated across three critical phases, explicitly addressing the operational gaps of intermittent connectivity and the lack of deterministic latency.

Phase A: Safety-critical event detection and handshake. The process begins in the Sensing Layer (Layer 1), where onboard sensors detect a hazard. To ensure immediate prioritization, the data is encapsulated into a Basic Safety Message (BSM) tagged with SAE J2735 metadata and a Priority=CRITICAL flag before being passed to the Communication Layer (Step 1). Simultaneously, ensuring the “handshake” required for resiliency, the Communication Layer (Layer 2) continuously reports real-time network health via the reportMetrics interface to the Monitoring Layer (Step 2). In this specific scenario, the interface reports metrics indicating congestion (e.g., latency of 120 ms, packet delivery ratio less than 85%).

Phase B: Survivability decision (the core logic). The intelligence driving the architecture resides in the Monitoring Layer (Layer 4). Upon receiving the metrics, an embedded Finite State Machine (FSM) evaluates the ‘Resiliency Index’ (*R_i_*) against pre-defined safety thresholds. Since the reported latency exceeds the safety margin (*L_e_*_2_*_e_* > 100 ms) (Step 3), the FSM triggers a ‘Degradation Interrupt’. This control signal (Step 4) is immediately sent to the Communication Layer, overriding standard routing tables to enforce the ‘Survivability Mode’. Consequently, the Communication Layer executes a service degradation routine (dropQueues), purging non-critical infotainment traffic (Step 5) to free up channel resources immediately.

Phase C: Resilient execution. With the channel cleared, the Communication Layer executes the transmission. Instead of relying on the congested primary interface, it switches to a redundancy-oriented protocol (e.g., Geocast or C-V2X Sidelink) to maximize reachability (Step 6). The message reaches the Processing Layer (Layer 3), hosted on a Multi-access Edge Computing (MEC) node, to minimize the number of hops. Here, the architecture validates data integrity using digital signatures and, upon successful verification, returns a signed acknowledgment (ack) to close the loop (Step 7).

Data consistency and security context. To support this high-speed decision-making, the data flow utilizes a Hierarchical Metadata Tagging scheme: as the packet moves from Layer 1 to Layer 3, tags such as {Time, Location, TrustScore, UrgencyLevel} remain as unencrypted headers. This allows intermediate nodes to perform the fast filtering seen in Phase B without needing to decrypt the full payload. Furthermore, the separation of the control plane (Monitoring) from the data plane (Communication) ensures that the heavy analytical tasks of the FSM do not impede the packet transmission, effectively mitigating the instability of vehicular links during the hazard.

### 4.6. Theoretical Validation and Asymptotic Complexity Analysis

Given that this work presents a reference architecture derived from a systematic review, we validate the proposed framework through asymptotic complexity analysis and architectural modeling rather than stochastic simulation. This approach allows us to determine the theoretical upper bounds of latency and overhead, demonstrating the architecture’s viability in critical worst-case scenarios, such as high-density broadcast storms, where traditional ad hoc protocols typically fail. By decoupling the control plane (Monitoring Layer) from the data plane, we demonstrate below how the proposed architecture reduces the computational complexity compared to standard reactive protocols.

#### 4.6.1. Impact of SDN on Routing Latency

Traditional reactive routing protocols (e.g., AODV) incur a route discovery latency proportional to the network diameter, *O*(*N*), which becomes prohibitive in dynamic topologies where links break frequently. By decoupling the control plane from the data plane, our architecture shifts the routing complexity to the Processing Layer (Edge/MEC). The SDN controller maintains a global view of the topology, allowing for proactive flow rule installation.

Consequently, the vehicle (data plane) simply forwards packets based on pre-cached flow tables, reducing the forwarding latency to a constant time lookup, *O*(1), per hop. Previous studies [55,100] have demonstrated that this hierarchical SDN approach maintains end-to-end latency below 50 ms even under frequent topology changes, satisfying the safety-critical requirements for collision warnings.

Analytically, the total latency (*T_total_*) in a connected car safety application can be modeled as the sum of transmission delay (*T_trans_*), propagation delay (*T_prop_*), and processing delay (*T_proc_*). In traditional cloud-centric architectures, *T_proc_* includes the Round-Trip Time (RTT) to the core network backhaul (*T_backhaul_*). Thus:

$$T_{cloud}=T_{trans}+T_{prop}+T_{backhaul}+T_{cloud\_compute}$$

In contrast, our multi-layered architecture utilizes the Processing Layer at the edge (MEC) and the Sensing Layer’s immediate survivability triggers. By eliminating *T_backhaul_* for safety-critical messages (as defined in our Cross-Layer protocol in Section 4.5), the latency becomes:

$$T_{proposed}=T_{trans}+T_{prop}+T_{edge\_compute}$$

Considering that *T_backhaul_* in LTE/5G non-standalone networks often ranges between 30 ms and 100 ms depending on congestion, and *T_edge_compute_* is negligible (less than 5 ms), our architecture theoretically guarantees adherence to the strict 10–20 ms safety threshold required for pre-crash sensing, which cloud-centric models mathematically cannot guarantee under high traffic load.

#### 4.6.2. Clustering Efficiency and Overhead Reduction

In a dense urban intersection, a standard V2V ‘flooding’ mechanism exhibits a message complexity of *O*(*N*^2^), where *N* is the number of vehicles, leading to rapid channel saturation (Broadcast Storm). Our architecture mitigates this through the Monitoring Layer’s context-aware filtering. By utilizing the ‘Survivability’ criteria defined in Section 3.5, the system dynamically isolates the broadcast domain to only *K* relevant nodes (those within the collision trajectory), where *K* is much lower than *N*.

Consequently, the signaling overhead is reduced from *O*(*N*^2^) to *O*(*K*) or quasilinear *O*(*K log K*) complexity depending on the clustering algorithm used. This reduction demonstrates that the architecture remains stable and survivable even as the total vehicle density *N* increases beyond the capacity of traditional IEEE 802.11p/DSRC channels.

#### 4.6.3. Comparative Qualitative Analysis

To validate the architectural design in the absence of field trials, we conducted a qualitative comparison against existing state-of-the-art frameworks. While the proposed multi-layered architecture offers superior theoretical performance in latency and resilience, it is essential to acknowledge the trade-offs regarding implementation complexity. Unlike ‘Pure Ad hoc’ solutions that require no infrastructure, our proposal relies on the deployment of Edge/MEC nodes (Processing Layer) and the synchronization of a Monitoring Layer. This introduces a ‘Deployment Cost’ and ‘Orchestration Overhead’ that are higher than those of simple reactive protocols. However, as Table 4 summarizes, this overhead is the necessary cost to achieve the ‘Survivability’ attribute, guaranteeing critical service continuity, which purely ad hoc or purely cloud-based approaches cannot robustly ensure in dynamic topologies.

As detailed in Table 4, unlike purely SDN-based approaches which suffer from single-point-of-failure risks, or purely MEC-based solutions that lack global coordination, our multi-layered approach provides a balanced trade-off. Specifically, by decoupling the control plane (Monitoring Layer) from the data plane (Communication Layer), our architecture theoretically minimizes the signaling overhead observed in mesh-based VANETs, offering superior scalability for high-density scenarios.

## 5. Conclusions

In this paper, we discussed the communication failure issues that may arise in the connected car ecosystem from network, hardware, software, and security perspectives. We considered these failures in the connected car environment. We reviewed the literature on recent works addressing specifically resiliency for connected cars to ensure uninterrupted telecommunication services on the road, such as infotainment, roadside assistance, and road user presence alerts. However, to maintain the continuous provision of these services, we must address several challenges, such as environmental issues, interference, and connectivity gaps. To solve these challenges, we must develop mechanisms that detect abnormal operations and allow immediate recovery to minimize network and user impacts. Additionally, it is crucial to integrate redundancy mechanisms and enhanced protocols to provide better network coverage and establish protection against electromagnetic interference and security breaches in user information.

The four-layer infrastructure proposed in this article (sensing, communication, processing, and monitoring) enables the collection of environmental sensing data from the vehicular environment to monitor potential failures and quickly adapt to changing information flow conditions. Based on this flow, we can ensure reliable data exchange among all road users, thereby enabling secure, resilient, and adaptive communication in the connected car environment. Additionally, the processing layer analyzes the information and prioritizes storage and availability transparently for users. Finally, the monitoring layer manages and oversees the services and infrastructure within the environment to respond efficiently and appropriately to real-time issues.

Finally, we recognize that, to comprehensively evaluate the impact of the proposed solutions, a dedicated experimental testbed is required. As we mentioned before, though this work validates the architecture through qualitative and theoretical analysis, the implementation of a physical testbed to evaluate the architecture’s effectiveness under real-world communication constraints is beyond the scope of this article but will be developed as part of our future work.

We believe that integrating resilience mechanisms and protocols in a connected car environment will enable the system to function optimally, providing the necessary services and infrastructure with the least interruption for safe, efficient, ubiquitous, and democratized interactions for all users in the connected vehicular environment.

## Figures and Tables

**Table 1:** Summary of the benefits of connected vehicles in the urban transport environment.

Benefit	Description
Greater road safety	The integration of communication technologies enables the exchange of information with other cars and the infrastructure, allowing them to work collaboratively to detect hazardous situations in real time, thereby reducing the likelihood of accidents.
Traffic optimization	The exchange of relevant data (location, speed, and destination) will facilitate better traffic management, optimize routes, and efficiently coordinate vehicle flow.
Improved user experience	Connected cars will offer different services, including advanced navigation, entertainment, and automated assistance, enhancing the user experience.
Energy savings	Connected cars will help reduce fuel consumption by better managing vehicle flow.
Sustainability and environment	Connected cars will contribute to reducing pollutant (CO_2_) emissions by optimizing routes, avoiding traffic congestion, and providing a more efficient and enjoyable driving experience.
Transportation as a Service (TaaS)	Connected cars can be integrated into shared mobility ecosystems (car sharing, ride sharing, on-demand transportation), providing real-time data that promotes collaborative and multimodal transportation, where the car is no longer an isolated asset but becomes part of the urban network.

**Table 2:** Challenges to deploy resilient communications and optimize traffic operations.

Challenge	Relevance	Causes	Consequences
Latency	Many systems and applications for vehicle safety require minimal delays because even a delay of 10–50 millisecond can make the difference between avoiding an accident and a collision.	Non-optimized communication protocols. Network congestion. Inefficient algorithms.	Delayed system responses. Road accident risks.
Scalability	The scalability of V2X communication introduces technical challenges: under high vehicle density, the radio channel can saturate; additionally, spectrum contention in IEEE 802.11p [17] and C-V2X can exacerbate network congestion. This calls for spectrum management, resource allocation, and traffic prioritization mechanisms.	Infrastructure constraints across roadway elements, communications (V2X/backhaul), and edge/compute capacity. Inadequate protocols for prioritizing safety-critical data flows (e.g., signal preemption, incident alerts), causing contention and delay.	V2X communication failures. Network performance degrades under heavy traffic.
Dynamic topology	High mobility leads to highly dynamic topologies and short-lived links, requiring adaptive protocols (neighbor discovery, routing, and handover) to preserve service continuity.	High node mobility, fluctuating vehicle density, frequent topology changes, and link churn. Coverage gaps and degraded radio conditions (load, Non-Line-Of-Sight (NLOS)).	Message loss and latency spikes. Unstable routing (route flaps, stale paths, and inconsistent next hops). Weak session persistence.
Data integrity	Data produced by vehicles and the roadside infrastructure may be inconsistent or untrustworthy due to sensor faults, packet loss, malicious tampering, or source heterogeneity. Therefore, distributed verification and consensus schemes (e.g., blockchain over C-V2X) are needed to ensure integrity, traceability, and non-repudiation.	Partial state replication (inconsistent views across edge and RSUs). Poor data handling.	Faulty application outputs. Erroneous control decisions. User privacy exposure.

**Table 3:** Key aspects and their description that highlight the importance of resiliency in the connected car domain.

Aspect	Description
Safety-critical systems continuity	Connected cars are based on software-controlled systems; thus a resilient design must preserve safety-critical functions such as braking and collision avoidance, even when portions of the network or sensors fail.
Privacy and data protection	The information exchanged between elements in the environment is highly significant for all involved. Resilience must implement measures and mechanisms to protect the driver’s privacy and prevent unauthorized access to information exchanged.
Infrastructure integration	The success of this environment requires integrating the connected car network and road infrastructures. Resilience should ensure the safety, reliability, and efficiency of interactions between these infrastructures, minimizing the risk of traffic issues.
Adaptability	The connected car environment is highly dynamic and requires resilience to ensure that cars adapt and respond to changes while maintaining peak performance.

**Table 4:** Comparative analysis of the proposed architecture against existing frameworks.

Comparison Criteria	Cloud-Centric Architectures [15]	Pure SDN-Based Approaches [55]	Pure MEC-Based Approaches [29]	Proposed Multi-Layer Architecture
Latency management	High (round-trip time > 100 ms). Dependent on the core network backhaul.	Medium. Control plane overhead can delay routing decisions.	Low. Processing at the edge, but potential handover delays.	Ultra-Low. Hybrid approach using direct Layer 2 switching and Edge processing.
Scalability	Low. Prone to bottlenecks at the central server.	Medium. Controller saturation risks in high-density scenarios.	High. Distributed processing but complex orchestration.	High. Hierarchical clustering reduces signaling overhead (*O*(*N log N*)).
Dynamic topology	Poor. Slow convergence: routing tables become stale.	Good. Global view allows adaptation but sensitive to controller link loss.	Medium. Localized view limits optimization of long-range paths.	Robust. Layer 2 redundancy and Layer 4 predictive monitoring handle rapid changes.
Data integrity	Centralized trust (single point of failure).	Vulnerable to control plane attacks (e.g., saturation).	Distributed trust but synchronization issues.	Hybrid. Real-time ECDSA verification and asynchronous Blockchain auditing.
Privacy support	Low. User data is aggregated centrally.	Medium. Flow rules may reveal trajectory patterns.	High. Data stays local but lacks global accountability.	High. Zero-Knowledge Proofs and local processing minimize data exposure.
Failure recovery	Slow. Relies on full system restart or backup.	Fast (control plane). Data plane recovery depends on new flow rules.	Fast (local). Isolation of failed nodes is efficient.	Resilient. “Survivability” mode maintains critical safety services during recovery.

## Data Availability

No new data were created or analyzed in this study.
